# An open-label, dose-escalation, safety, and pharmacokinetics phase I study of ombrabulin, a vascular disrupting agent, administered as a 30-min intravenous infusion every 3 weeks in Japanese patients with advanced solid tumors

**DOI:** 10.1007/s00280-014-2388-x

**Published:** 2014-01-30

**Authors:** H. Murakami, T. Kurata, Y. Onozawa, J. Watanabe, A. Ono, T. Takahashi, N. Yamamoto, Y. Fujisaka, H. Kiyota, H. Hayashi, K. Tanaka, K. Nakagawa, S. Kuroda

**Affiliations:** 1 Division of Thoracic Oncology, Shizuoka Cancer Center, 1007, Shimonagakubo Nagaizumi-cho Sunto-gun, Shizuoka, 411-8777 Japan; 2 Department of Medical Oncology, Faculty of Medicine, Kinki University, Osaka, Japan; 3 Division of Gastrointestinal Oncology, Shizuoka Cancer Center, Shizuoka, Japan; 4 Division of Breast Oncology, Shizuoka Cancer Center, Shizuoka, Japan; 5 Research and Development, Sanofi K.K., Tokyo, Japan

**Keywords:** Japanese patients, Ombrabulin, Pharmacokinetic, Phase I, Vascular disrupting agent

## Abstract

**Purpose:**

To determine ombrabulin’s maximum tolerated dose and dose recommended for Japanese patients with advanced solid tumors and to assess its antitumor activity and overall safety and pharmacokinetic profiles.

**Methods:**

This was a multi-center, open-label, sequential-cohort, dose-escalation phase I study of ombrabulin, a vascular disrupting agent, administered once every 3 weeks. Patients were treated with 15.5, 25, 35, or 50 mg/m^2^ ombrabulin over a 30-min intravenous infusion. The recommended dose was the highest dose at which <33 % of all evaluable patients experienced dose-limiting toxicities (DLTs) during the first treatment cycle or 50 mg/m^2^ (recommended in Caucasian patients) if the previous definition was not met.

**Results:**

Fifteen patients were treated. No DLT occurred with 15.5, 25, or 35 mg/m^2^ ombrabulin. In the 50 mg/m^2^ group, one patient had Grade 3 lymphopenia, and another experienced Grade 2 hypertension and Grade 3 diarrhea judged as DLTs. The most frequent related adverse events in this group were diarrhea, nausea, and hypertension. Two patients had Grade 3 anemia, one at the 15.5 mg/m^2^ and the other at the 50 mg/m^2^. No AEs necessitating dose reduction or Grade 4 AEs were observed. Overall, five patients had stable disease. Pharmacokinetic parameters were comparable to those in non-Japanese patients.

**Conclusions:**

Ombrabulin treatment once every 3 weeks was well tolerated in Japanese patients with advanced solid tumors. The dose recommended is 50 mg/m^2^, as in Caucasian patients. The safety and pharmacokinetic profiles were comparable between Japanese and Caucasian patients (funded by Sanofi; ClinicalTrials.gov number, NCT00968916).

## Introduction

The concept that tumor growth and survival are critically dependent on the development of new blood vessels has led to the development of therapeutics that target angiogenesis [1]. There are currently two principal mechanisms by which vascular-targeted agents exert their effects: inhibition of angiogenesis (antiangiogenic agents) and destruction of the existing tumor vasculature (vascular disrupting agents, VDAs) [2]. By virtue of their high proliferation rate, endothelial cells of tumor blood vessels are far more sensitive to VDAs than are cells of normal blood vessels [3]. On the molecular level, ombrabulin, one such VDA, binds to the colchicine site of tubulin and inhibits polymerization, causing destabilization of the cytoskeleton, shape change, and vascular collapse [4, 5]; the resultant reduction in blood flow leads to tumor necrosis. VDAs have demonstrated varying degrees of success in the clinical setting. Colchicine was the first tubulin-binding agent discovered to have antivascular effects, which resulted in hemorrhagic necrosis in human tumors [6]. However, its toxicity prevented further clinical evaluation. Tubulin-binding agents such as vincristine and vinblastine are potent anticancer drugs currently used. These agents induce extensive vascular damage in animal tumors at doses close to their respective maximum tolerated doses (MTDs) [7, 8]. Despite these advances, more potent VDAs are currently being sought.

**Fig. 1:**
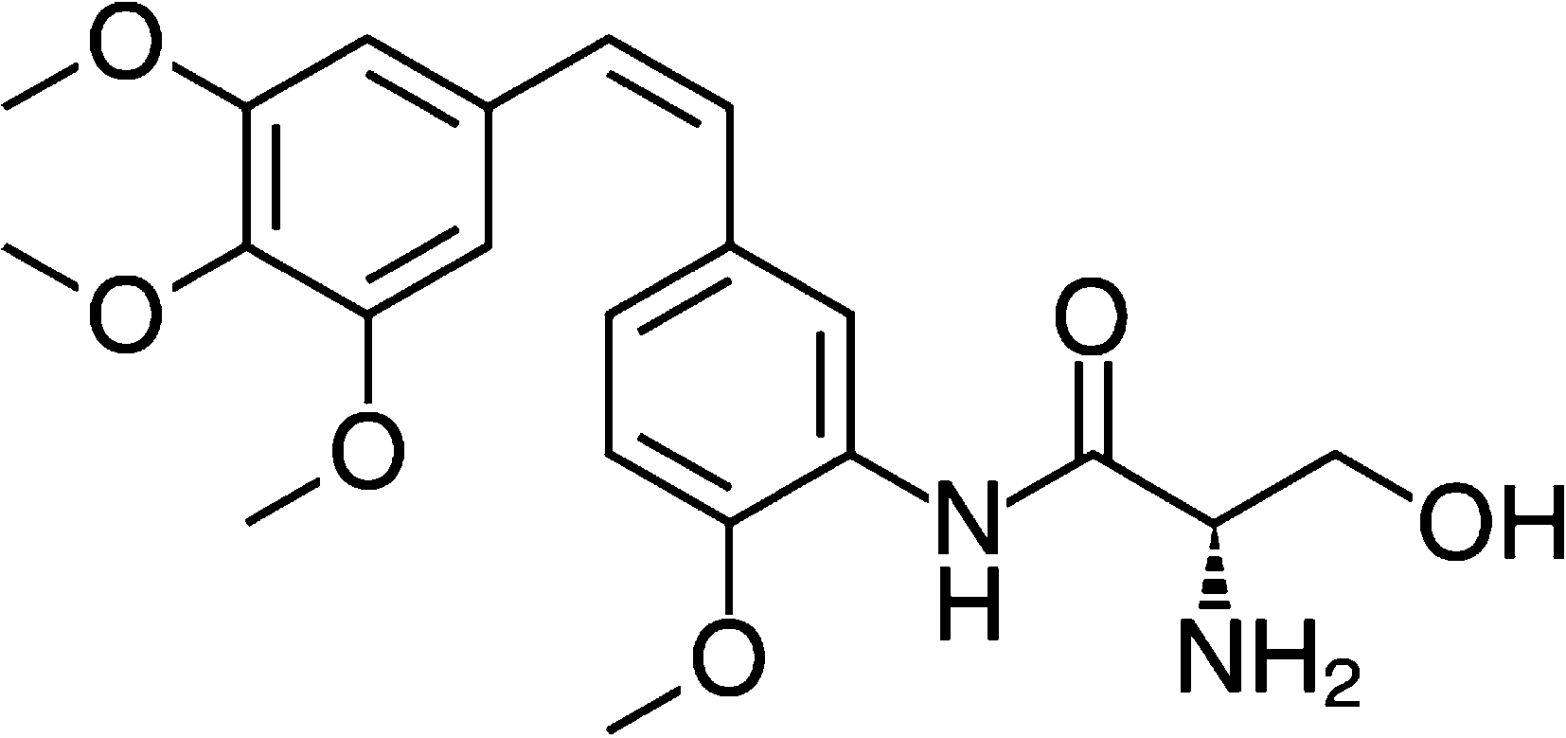
Chemical structure of ombrabulin (AVE8062)

Combretastatins are a class of compounds isolated from the South African tree *Combretum caffrum* that have a high affinity for tubulin at or near the colchicine-binding site. Combretastatin A4 (CA-4) [9], in particular, is highly cytotoxic [10, 11]. Combretastatin A4 phosphate (CA4P), a prodrug of CA-4, and ombrabulin (AVE8062), a synthetic analog of CA4P (Fig. 1), have been shown to suppress tumor growth by reducing tumor blood flow in different cancers in vivo [12–15]. In mice, ombrabulin began to restrict tumor perfusion within 15 min after the start of infusion; peak effect occurred after 6 h, with permanent widespread tumor necrosis after 24 h [16]. As with other VDAs, perfusion changes seen in normal tissues were largely reversible [17], indicating that tumor tissue is more susceptible to ombrabulin-induced reduction in blood flow [18]. Results from toxicological studies suggest that ombrabulin mainly affects cells with high turnover, including bone marrow, circulating blood cells, and intestinal epithelia [3]. However, cardiovascular effects, including myocardial degeneration and necrosis, hypertension, and premature ventricular contractions, were also noted with high-dose ombrabulin in different species [3].

The recommended dose derived from a phase I dose-escalation study conducted in Europe, in which ombrabulin was administered once every 3 weeks over a dose range of 6–60 mg/m^2^, was 50 mg/m^2^ [19]. On the basis of these results, we conducted a new phase I study designed to evaluate DLTs and MTD for Japanese patients with advanced solid tumors and to assess pharmacokinetics and safety profiles between Japanese and Caucasian patients.

## Patients and methods

### Study conduct

The study was approved by the institutional review boards/ethics committees and was conducted in accordance with the Declaration of Helsinki. All patients gave written informed consent prior to enrollment.

### Patient selection

Adult Japanese patients with advanced histologically or cytologically proven solid malignant tumors refractory to conventional treatment or without standard therapeutic options were eligible. The main inclusion criteria were the following: (1) age ranging from 20 to 75 years, (2) Eastern Cooperative Oncology Group (ECOG) performance status of <2, and (3) life expectancy of more than 12 weeks. The main exclusion criteria were as follows: (1) concurrent treatment with any other anticancer therapy, including chemotherapy, immunotherapy, radiotherapy, targeted therapy, or gene therapy, (2) washout period of <28 days from prior anticancer therapies (chemotherapy, targeted agents, immunotherapy, and radiotherapy) or any investigational treatment, (3) symptomatic brain metastases and carcinomatous leptomeningitis, (4) inadequate organ function including neutrophil count of <1.5 × 10^9^/L, platelet count of <100 × 10^9^/L, hemoglobin level of <9.0 g/dL, creatinine clearance of <60 mL/min, total bilirubin level of ≥1.5 mg/dL, alanine aminotransferase (ALT) or aspartate aminotransferase (AST) level of >1.5 times the upper limits of normal (ULN) of the institutional norms, and alkaline phosphatase (AP) level of >2.5 times ULN of the institutional norms, (5) a left ventricular ejection fraction (LVEF) of <50 % by echocardiography, (6) a baseline QTc interval of >0.45, (7) hypertension defined as systolic blood pressure (SBP) of >140 mmHg or diastolic BP (DBP) of >90 mmHg, and (8) 1 or more episodes of ventricular tachycardia with 3 or more consecutive premature beats with a frequency of ≥180 beats/min.

### Study design

This was a multi-center, open-label phase I study designed to test the safety and pharmacokinetics (PK) of ombrabulin (Sanofi Oncology, Vitry-sur-Seine, France) in Japanese patients with advanced malignant solid tumors (ClinicalTrials.gov: NCT00968916). The primary objective was to determine the maximum tolerated dose (MTD) on the basis of the incidence of dose-limiting toxicity (DLT) during Cycle 1. Ombrabulin was administered as a 30-min IV infusion once every 3 weeks to sequential cohorts of three patients at a dose 15.5, 25, 35, or 50 mg/m^2^; the study design allowed for the addition of three patients if one patient experienced a DLT at that dose. The decision to proceed to the next dose was primarily based on the identification of DLTs. The dose escalation was discontinued when two or more of six patients experienced a DLT; the dose at which this occurred was designated the maximum administered dose (MAD), and additional patients were to be enrolled in the previous dose level (the MTD) until at least six patients could be evaluated. The starting dose and dose-escalation increments were based on results from previous trials [19–21] to allow for a direct comparison of results with Japanese versus Caucasian patients. Because this is the first phase I trial in Japanese patients, the starting dose of 15.5 mg/m^2^ was selected since no DLTs had been reported at this dose level in monotherapy and combination studies, and imaging studies showed evidence of tumor blood flow shutdown [20]. The MTD for ombrabulin monotherapy with a triweekly schedule was previously established at 50 mg/m^2^ [19]; thus, 50 mg/m^2^ was the highest dose used in the present study. Although the results suggest that this is not the MTD for Japanese patients, the dose range was not extended to identify the MTD, and the study was completed.

### Safety evaluations

The safety population was to be the all enrolled patients who received at least 1 (even if incomplete) infusion of ombrabulin. AEs were graded according to the National Cancer Institute Common Terminology Criteria (NCI-CTC) version 3.0. Hematology/biochemistry and urinalysis was evaluated weekly. Cardiovascular evaluations included a clinical examination, 12-lead and 24-h Holter ECGs, and plasma levels of creatine phosphokinase (CPK), CK-MB, and troponin I. Echocardiography, chest X-ray, and brain MRI were performed every other cycle from the second cycle. To qualify as a DLT, clinical AEs or laboratory abnormalities had to be drug-related as assessed by the investigator during cycle 1. Non-vascular hematologic DLTs were defined as follows: Grade 4 febrile neutropenia, Grade 4 neutropenia lasting >5 days, Grade 4 thrombocytopenia (platelet counts of <25 × 10^9^/L), or hemoglobin level of <6.5 g/dL. Non-vascular non-hematologic DLTs were defined as any Grade 3–4 events except nausea; vomiting well controlled under antiemetic treatment; increases in AST, ALT, or AP levels lasting <8 days; or hypersensitivity well controlled under antihistamine and corticosteroid treatment. Non-vascular renal DLTs were defined as calculated creatinine clearance of <40 mL/min. Cardiovascular DLTs were defined as follows: documented angina pectoris or arterial thromboembolism; hypertension with systolic BP of ≥180 mmHg or diastolic BP of ≥120 mmHg for at least 2 successive measurements, or acute impairment of target organ (the brain, heart, and kidney); hypotension grade of ≥2 or systolic BP of <90 mmHg for at least 2 successive measurements; positive cardiac markers with troponin-I value greater than the pathological limit defined by the manufacturer as a sign of myocardial necrosis, cardiac ischemia/infarction with ST- and T-wave changes suggesting ischemia; decreased cardiac left ventricular function with resting ejection fraction below 50 % with ≥20 % decline of resting ejection fraction from baseline value; ventricular arrhythmia accompanied by at least 1 series of ventricular tachycardia with 3 or more consecutive premature beats, with a frequency of ≥180 beats/min; or any other Grade 3–4 vascular events, except for some adverse events at tumor site, such as intratumor hemorrhage or tumor hemorrhagic necrosis, that are not life-threatening. A patient was to be considered as evaluable for a DLT if the patient had received at least one administration of ombrabulin and if a complete safety evaluation had been performed during Cycle 1.

### Tumor response evaluation

The efficacy endpoint in evaluable patients was the objective tumor response as defined by the rules set forth in the Response Evaluation Criteria in Solid Tumors (RECIST 1.0) [22]. Patients with an objective response were defined by pooling those who exhibited a complete response (CR) or a partial response (PR). Tumor assessments of all target or non-target lesions and brain magnetic resonance imaging (MRI)/computed tomography (CT) were performed within 28 days of first dose administration. Subsequent assessments were performed every 2 cycles until unacceptable disease progression or treatment discontinuation.

### Pharmacokinetics

Plasma samples were obtained from all patients before infusion, immediately prior to the end of the 30-min infusion (EOI), and at 5, 10, 25, 45, and 60 min, and 2, 4, 6, 10, 24, and 48 h after EOI at Cycle 1. During Cycles 2–4, samples were collected immediately prior to the EOI and 10 min after infusion. Concentrations of ombrabulin and its active metabolite RPR258063 were measured by LC–MS/MS. The limit of quantification (LOQ) was 2.00 ng/mL for both analytes. All PK parameters, including maximum plasma concentration (C_max_), area under the curve (AUC), terminal half-life (T_1/2z_), clearance (CL), and volume of distribution at steady state (VD_ss_), were calculated by non-compartmental analysis using WinNonlin software (version 5.2.1). Pharmacokinetic parameters were analyzed using descriptive statistics, including mean and standard deviation. Statistical modeling was used to assess dose proportionality of C_max_ and AUC.

## Results

### Patient characteristics and treatment

Overall, 15 Japanese patients were recruited and treated in two investigational centers in Japan from September 2009 to August 2011. There were no important deviations to the protocol. Three patients each were exposed to ombrabulin at a dose of 15.5, 25, or 35 mg/m^2^; six patients were exposed to 50 mg/m^2^ ombrabulin. One patient (6.7 %) discontinued study treatment due to diarrhea and hypertension. During the study, disease progression was observed in 14 patients (93.3 %), and no deaths were reported. Baseline characteristics of nine female (60.0 %) and six male patients (40.0 %) are summarized in Table 1. The treated patients had a performance status (PS) of 0 (46.7 %) or 1 (53.3 %) at baseline. The median (range) age was 62 (29–71) years, with most patients in the 50–64 years of age bracket (46.7 %). Almost all patients had metastatic tumors at baseline. The most frequent primary tumor sites were lung (6 of 15 patients), muscle/soft tissue (2 of 15 patients), and breast (2 of 15 patients). The median number of cycles was 2.0 (range 1–7) for doses ≤35 mg/m^2^ and 2.5 for the 50 mg/m^2^ dose.

**Table 1:** Patient baseline characteristics

*N* patients treated	Dose level (mg/m^2^)				Total
	15.5	25	35	50	
	3	3	3	6	15
Male, *N* (%)	1 (33.3 %)	1 (33.3 %)	1 (33.3 %)	3 (50.0 %)	6 (40.0 %)
Age in years, median (range)	60.0 (57–63)	67.0 (62–71)	61.0 (45–62)	63.0 (29–69)	62.0 (29–71)
Number of previous regimens, median (range)	5.0 (3–5)	5.0 (3–11)	0.0 (0–9)	2.5 (0–9)	3.0 (0–11)
Prior radiotherapy	2 (66.7 %)	2 (66.7 %)	2 (66.7 %)	1 (16.7 %)	7 (46.7 %)
Prior surgery	1 (33.3 %)	2 (66.7 %)	3 (100 %)	4 (66.7 %)	10 (66.7 %)
Prior chemotherapy	3 (100 %)	3 (100 %)	2 (66.7 %)	5 (83.3 %)	13 (86.7 %)
Prior hormonotherapy	0	1 (33.3 %)	1 (33.3 %)	0	2 (13.3 %)
Other prior therapies	0	0	0	1 (16.7 %)	1 (6.7 %)
ECOG performance status, *N* (%)					
0	0	1 (33.3 %)	2 (66.7 %)	4 (66.7 %)	7 (46.7 %)
1	3 (100 %)	2 (66.7 %)	1 (33.3 %)	2 (33.3 %)	8 (53.3 %)
Primary tumor, *N* (%)					
Lungs	3 (100 %)	1 (33.3 %)	1 (33.3 %)	1 (16.7 %)	6 (40.0 %)
Muscle/soft tissue	0	0	0	2 (33.3 %)	2 (13.3 %)
Breast	0	1 (33.3 %)	1 (33.3 %)	0	2 (13.3 %)
Head/neck	0	1 (33.3 %)	0	0	1 (6.7 %)
Esophagus	0	0	0	1(16.7 %)	1 (6.7 %)
Other^a^	0	0	1 (33.3 %)	1 (16.7 %)	2 (13.3 %)
Thyroid	0	0	0	1 (16.7 %)	1 (6.7 %)
Extent of disease at study entry, *N* (%)					
Metastatic	3 (100 %)	3 (100 %)	3 (100 %)	5 (83.3 %)	14 (93.3 %)
Locally advanced	0	0	0	1 (16.7 %)	1 (6.7 %)

^a^Malignant mesothelioma and adenocarcinoma of unknown primary

### Dose-limiting toxicity

The primary safety variable was the incidence of DLTs at Cycle 1. No DLTs occurred at 15.5, 25, or 35 mg/m^2^. One patient of six from the 50 mg/m^2^ treatment group experienced two incidences of DLT: Grade 2 hypertension, which occurred at the end of infusion and resolved that day, and Grade 3 diarrhea, which occurred during the subsequent 2 days and resolved the next day. The MTD defined in the protocol was not identified. However, since a dose higher than the global recommended dose (50 mg/m^2^) could not be tested, further dose escalation was not investigated. The dose level of 50 mg/m^2^ ombrabulin was therefore considered as the MAD and recommended dose (RD)/MTD in Japanese patients with advanced solid tumors.

### Overall safety

All patients experienced non-serious AEs; 14 of 15 patients experienced AEs considered related to study treatment. The most common AEs are summarized in Table 2. Overall, there were no AEs leading to death, no serious adverse events (SAEs) or those leading to dose reduction, no Grade 4 AEs, and no Grade 4 clinical laboratory abnormalities. In addition, no severe myelotoxicity or abnormal increase in cardiac markers was observed. Grade 1–2 neutropenia was observed in 8 of 15 patients (53 %) across all dose groups. Anemia was present in 10 of 15 (67 %) patients at baseline; after receiving ombrabulin, most patients (12 of 15) had anemia, including one patient each at 15.5 and 50 mg/m^2^ at Grade 3. There were no Grade 3–4 abnormalities in liver or renal functions. The most common AEs at 50 mg/m^2^ (RD) were diarrhea (five of six patients), nausea (five of six patients), and hypertension (four of six patients); 50 % of patients experienced decreased appetite, headache, conjunctival hyperemia, sinus bradycardia, hot flush, vomiting, or fatigue. One patient exposed to 50 mg/m^2^ had treatment-related Grade 2 and Grade 3 LV dysfunction (LVEF: 37 %) at Cycles 5 and 7, respectively.

**Table 2:** The most common adverse events observed in at least two patients at 50 mg/m^2^

	Dose level (mg/m^2^), *N* (%)							
	15.5 (*N* = 3)		25 (*N* = 3)		35 (*N* = 3)		50 (*N* = 6)	
	All grades	Grades 3 and 4	All grades	Grades 3 and 4	All grades	Grades 3 and 4	All grades	Grades 3 and 4
Patients with any AE	3 (100 %)	0	3 (100 %)	0	3 (100 %)	0	6 (100 %)	3 (50.0 %)
Patients with any related AE	3 (100 %)	0	2 (66.7 %)	0	3 (100 %)	0	6 (100 %)	2 (33.3 %)
Patients with any serious AE	0	0	0	0	0	0	0	0
AE (PT: alphabetical order)								
Blood bilirubin increased	0	0	0	0	0	0	2 (33.3 %)	0
Chills	0	0	0	0	0	0	2 (33.3 %)	0
Conjunctival hyperemia	0	0	0	0	0	0	3 (50.0 %)	0
Constipation	1 (33.3 %)	0	1 (33.3 %)	0	0	0	1 (16.7 %)	0
Decreased appetite	2 (66.7 %)	0	1 (33.3 %)	0	1 (33.3 %)	0	3 (50.0 %)	0
Diarrhea	1 (33.3 %)	0	0	0	1 (33.3 %)	0	5 (83.3 %)	1 (16.7 %)
Fatigue	1 (33.3 %)	0	1 (33.3 %)	0	1 (33.3 %)	0	3 (50.0 %)	0
Headache	0	0	0	0	1 (33.3 %)	0	3 (50.0 %)	0
Hot flush	0	0	0	0	1 (33.3 %)	0	3 (50.0 %)	0
Hypertension	1 (33.3 %)	0	0	0	1 (33.3 %)	0	4 (66.7 %)	0
Injection site phlebitis	0	0	0	0	1 (33.3 %)	0	2 (33.3 %)	0
Nasopharyngitis	1 (33.3 %)	0	2 (66.7 %)	0	0	0	1 (16.7 %)	0
Nausea	2 (66.7 %)	0	0	0	2 (66.7 %)	0	5 (83.3 %)	0
Pyrexia	1 (33.3 %)	0	0	0	0	0	2 (33.3 %)	0
Sinus bradycardia	0	0	0	0	0	0	3 (50.0 %)	0
Ventricular extrasystoles	0	0	0	0	2 (66.7 %)	0	1 (16.7 %)	0
Vomiting	2 (66.7 %)	0	0	0	1 (33.3 %)	0	3 (50.0 %)	0

AE is defined as an adverse event that is reported during the on-treatment period (from the first dose to 30 days after the last dose)

*PT* preferred term (MedDRA 14.0 and graded using NCI CTC version 3.0)

### Pharmacokinetics

**Fig. 2:**
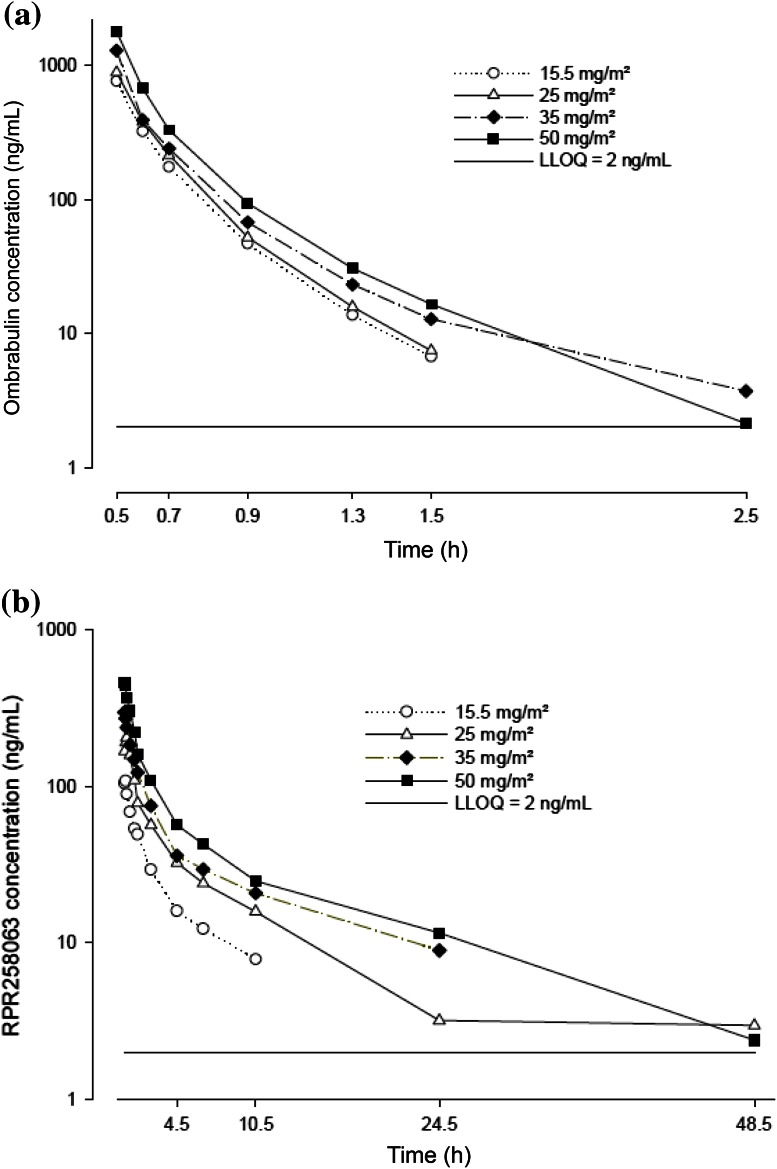
Mean plasma concentration–time profiles of ombrabulin and its major metabolite (RPR258063) in Cycle 1. **a** Ombrabulin, **b** RPR258063

PK analyses at Cycle 1 were performed on the PK-evaluable population by using actual sampling times, dosing times, and times of administration for each patient. Plots of the mean ombrabulin and RPR258063 plasma concentrations versus time profiles in patients for each ombrabulin dose level are shown in Fig. 2. Ombrabulin and RPR258063 PK parameters obtained at Cycle 1 are summarized in Table 3. After a single IV administration, maximum ombrabulin concentrations were observed at the end of infusion; concentrations rapidly declined and were quantifiable up to 1.5 or 2.5 h after administration. Ombrabulin was rapidly converted to RPR258063, the active metabolite, which appeared rapidly after end of infusion; exposure to the metabolite was found to be about 1.8-fold higher than to ombrabulin. T_1/2z_ (around 11 h) for RPR258063 was considerably longer in comparison with ombrabulin. Ombrabulin exhibited a high plasma CL (95.2 L/h) and a low VD_ss_ (30.9 L), corresponding to a short T_1/2z_ (15.7 min). Ombrabulin C_max_, AUC, and AUC_last_ did not deviate significantly from dose proportionality over the 15.5–50 mg/m^2^ dose range. RPR258063 exposure parameters were also dose proportional within this dose range. No dose effect was observed with T_1/2z_ or CL. No notable changes were observed in plasma concentration of ombrabulin and RPR258063 for cycles 1–4.

**Table 3:** Ombrabulin (a) and RPR258063 (b) pharmacokinetic parameters after single ombrabulin infusion

Ombrabulin dose	Plasma ombrabulin (mean ± SD)				Total
	15.5 mg/m^2^	25 mg/m^2^	35 mg/m^2^	50 mg/m^2^	
*N* patients treated	3	3	3	6	15
(a) Ombrabulin					
C_max_ (ng/mL)	766 ± 75.7	890 ± 322	1,300 ± 309	1,800 ± 334	NA
AUC (ng h/mL)	305 ± 38.8	443^a^	602^b^	750 ± 190	NA
t_1/2z_ (min)	12.6 ± 0.930	12.8^a^	17.6^b^	17.6 ± 4.31	15.7 ± 4.41
CL (L/h)	68.8 ± 8.59	97.1^a^	93.0^b^	108 ± 39.9	95.2 ± 31.3
VD_ss_ (L)	21.0 ± 2.52	33.2^a^	31.5^b^	34.9 ± 11.6	30.9 ± 9.59
(b) RPR258063					
C_max_ (ng/mL)	110 ± 35.5	192 ± 55.0	313 ± 20.2	478 ± 47.0	NA
AUC (ng h/mL)	345 ± 111	881 ± 251	1,020 ± 212	1,430 ± 307^c^	NA
t_1/2z_ (h)	7.49 ± 3.96	13.8 ± 6.70	12.0 ± 1.15	10.4 ± 3.21^c^	10.8 ± 4.22^d^
Metabolic ratio^g^ (AUC)	1.18 ± 0.551	1.85^e^	1.91^e^	2.14 ± 0.672^c^	1.81 ± 0.632^f^

NA, not applicable; C_max_, maximum plasma concentration observed; AUC, area under the concentration versus time curve extrapolated to infinity; t_1/2z_, terminal elimination half-life calculated using the following equation: t_1/2z_ = 0.693 × λ_z_; CL, total body clearance calculated using the following equation: CL = dose/AUC, for ombrabulin only; VD_ss_, volume of distribution at the steady state after single dose

^a^ *n* = 2, mean (t_1/2z_ not calculable)

^b^ *n* = 2, mean (t_1/2z_ not calculable)

^c^ *n* = 5 (t_1/2z_ not calculable)

^d^ *n* = 14

^e^ *n* = 2, mean

^f^ *n* = 12

^g^AUC(RPR258063)/AUC(ombrabulin)

### Antitumor activity

Tumor response (best overall response), assessed according to RECIST 1.0 criteria, revealed that 5 of 15 patients (33.3 %), including two patients with lung cancer, two patients with muscle/soft tissue cancer, and one patient with cancer of unknown primary origin, had stable disease (SD) at the end of the trial as assessed by the investigator. Sustained SDs longer than 4 months (maximum 7.2 months) were confirmed in two of five patients. Four of six patients (66.7 %) from the 50 mg/m^2^ dose group had SD, while among the nine patients in the lower dose groups (15.5, 25, and 35 mg/m^2^), one patient receiving a dose of 15.5 mg/m^2^ had SD (Table 4).

**Table 4:** Tumor responses

Best overall response [*n* (%)]	Dose level (mg/m^2^)				Total (*N* = 15)
	15.5 (*N* = 3)	25 (*N* = 3)	35 (*N* = 3)	50 (*N* = 6)	
Stable disease (SD)	1 (33.3 %)	0	0	4 (66.7 %)	5 (33.3 %)
Progressive disease (PD)	2 (66.7 %)	3 (100 %)	3 (100 %)	1 (16.7 %)	9 (60.0 %)
Not applicable/not assessed	0	0	0	1 (16.7 %)	1 (6.7 %)

## Discussion

Following the protocol used in an earlier phase I trial conducted in USA and Europe [19], we conducted a study in which Japanese patients were given ombrabulin once every 3 weeks at a dose of 15.5, 25, 35, or 50 mg/m^2^. No DLTs were observed up to 35 mg/m^2^. Only one of six patients treated at 50 mg/m^2^ experienced DLTs, including transient Grade 2 hypertension and Grade 3 diarrhea. Thus, the RD for Japanese patients was determined to be 50 mg/m^2^. Although we had not yet reached the MTD for Japanese patients at 50 mg/m^2^, the study was not extended to include higher doses since Sessa et al. [23] had determined the RD for phase II studies to be 50 mg/m^2^. In their study, one patient experienced DLT (Grade 3 abdominal pain) at a dose of 50 mg/m^2^, and another experienced Grade 3 tumor pain/Grade 3 hypertension at 60 mg/m^2^. Although adverse events were generally more common, the safety profile observed in our study is very similar to that in a study conducted in USA and Europe [19]. In that study, the most common drug-related adverse events at the RD of 50 mg/m^2^ were headache (31 %), asthenia (28 %), abdominal pain (26 %), nausea (26 %), diarrhea (23 %), and hypertension (23 %); these too were mainly mild to moderate [19].

Results compiled from early-phase clinical trials involving VDAs have demonstrated a profound occurrence of cardiovascular events, including hypertension, tachycardia, bradycardia, atrial fibrillation, and myocardial infarction [24]. In the present study, three patients with a medical history of hypertension (one patient from the 35 mg/m^2^ and two patients from the 50 mg/m^2^ groups) experienced SBP ≥20 mmHg increase from baseline and ≥180 mmHg within an hour of ombrabulin treatment; blood pressure returned to baseline levels on the same or next day without any corrective treatment. Similarly, in a phase I trial with ombrabulin, Sessa et al. [23] reported a 23 % incidence of mild to moderate hypertension that was rapid in onset and transient. In the one patient in whom we noted treatment-related LV dysfunction, it was not considered a serious event, even though it had been present for 5 months, because LVEF had been low (54 %) at baseline. Other reported cardiotoxicities include Grade 1–2 atypical chest pain with evidence of ischemia on ECG and transient QTc prolongation [24]. We did note one patient in the 50 mg/m^2^ treatment group with a Grade 1 increase in troponin I.

No dose effect was observed on terminal elimination half-life or clearance of ombrabulin or on terminal elimination half-life of RPR258063. The PK profiles for both ombrabulin and RPR258063 determined in our study were comparable to those obtained by Sessa et al. [19] in Caucasian subjects.

This was a phase I study, and tumor response was not a primary endpoint. No patient had a complete or partial response as the best response, although 5 of 15 patients (33.3 %) overall and four of six patients (66.7 %) from the highest dose group (50 mg/m^2^) had SD at the end of the trial. In the preceding phase I study in non-Japanese patients treated with 50 mg/m^2^ ombrabulin [19], a significant increase in circulating endothelial cells (CECs), a potential predictive/prognostic biomarker for antiangiogenic treatment efficacy [25, 26] and quantitative pharmacodynamic biomarker of VDA bioactivity [27], was reported in 11 out of 15 tested patients, potentially reflecting maximal acute vascular damage, and one patient showed partial response. This increase in CECs reported by Sessa et al. was probably due to variations in the timing of tumor response, and an optimal timing for use of this parameter should be explored.

In summary, a 30-min infusion of up to 50 mg/m^2^ ombrabulin administered to Japanese patients once every 3 weeks is well tolerated with limited cardiovascular adverse events. Therefore, 50 mg/m^2^ is considered the RD for Japanese patients with advanced solid tumors being treated with a single agent.
